# Screening and Identification of Hnf1ba-*slc12a1* Signal Pathway in Response to Low-Salinity Stress in Marine Medaka (*Oryzias melastigma*)

**DOI:** 10.3390/ijms262311402

**Published:** 2025-11-25

**Authors:** Binghua Liu, Lei Lin, Meng Wang, Jingjing Zhang, Yu Yang, Hong-Yan Wang, Changwei Shao

**Affiliations:** 1 State Key Laboratory of Mariculture Biobreeding and Sustainable Goods, Yellow Sea Fisheries Research Institute, Chinese Academy of Fishery Sciences, Qingdao 266071, China; liubh@ysfri.ac.cn (B.L.); linleicaas@163.com (L.L.); mandy001106@163.com (M.W.); jingjingzhang0307@163.com (J.Z.); yyu13156168974@163.com (Y.Y.); wanghongyan@ysfri.ac.cn (H.-Y.W.); 2 Laboratory for Marine Fisheries Science and Food Production Processes, Qingdao Marine Science and Technology Center, Qingdao 266237, China

**Keywords:** osmotic pressure regulation, *Oryzias melastigma*, Hnf1ba-*slc12a1* signal pathway, transcription binding site, CRISPR-dCas9 & Sun-Tag system, targeted regulation experiment

## Abstract

Euryhaline fishes provide excellent material for the theoretical study of the broad-spectrum adaptability of organisms and the use of low-salinity and even freshwater environments, or high-salinity and seawater environments, for the domestication of fishes. Here, we studied the molecular mechanisms of osmotic pressure regulation in a euryhaline fish, marine medaka (*Oryzias melastigma*). As the fish progressed from seawater to freshwater, the changes in stress indicators (cortisol—COR; malondialdehyde—MDA; reactive oxygen species—ROS; superoxide dismutase—SOD) indicated that they gradually adapted to the freshwater environment. The transcriptome analysis also showed that there were 6850 DEGs (differentially expressed genes) involved in the process. By analyzing these DEGs deeply, we screened and identified the Hnf1ba-*slc12a1* signal pathway involved in osmotic pressure regulation. The results of a dual-luciferase reporter assay in HEK293T cells, as well as an overexpression experiment by in vitro cultured gill cells of *O. melastigma*, confirmed that Hnf1ba transcriptionally regulates the *slc12a1* gene. Fragment deletion and site-directed mutagenesis assays revealed a Hnf1ba-binding sequence (GATTAATCATTTACT, located at −1877 to −1863) in the *slc12a1* promoter. Based on this result, we conducted a targeted regulation experiment on the *slc12a1* gene using the CRISPR-dCas9 & Sun-Tag system. The most effective activation of *slc12a1* gene expression was observed in the sgRNA2 group. These results enhance our understanding of adaptation mechanisms in salt-tolerant fish and provide a reference for efficiently promoting the domestication of fish adaptive to salinity changes.

## 1. Introduction

Salinity difference is a typical characteristic that distinguishes seawater from freshwater, and it dictates the ecological niches and survival strategies of organisms residing within these water environments. Aquatic organisms are often restricted to thriving and propagating solely within their respective salinity habitats, whether in the hypersaline water of seawater systems, hypo-osmotic water of freshwater systems, or brackish water of estuaries [1,2]. They usually have a limited tolerance range for salinity, and changes in salinity can disrupt their osmotic balance between the internal body fluids and the external environment. However, there are intriguing exceptions to this rule among a few species, which are able to transcend these salinity boundaries and adapt to both hyper-osmotic and hypo-osmotic conditions. They are called euryhaline fishes, such as spotted sea bass (*Lateolabrax maculatus*) [3], marine medaka (*Oryzias melastigma*) [4], Japanese eel (*Anguilla japonica*) [5], blackchin tilapia (*Sarotherodon melanotheron*) [6], and Mozambique tilapia (*Oreochromis mossambicus*) [7]. Some euryhaline fishes can even survive normally in high-salinity seawater and zero-salinity freshwater. This wide salt-tolerance adaptability offers a compelling research avenue for promoting the domestication of marine fish in low-salinity and even freshwater environments, or that of freshwater fish in high-salinity and even seawater environments. Domestication can provide access to a wider range of fish species for cultivation, thereby enhancing market opportunities and expanding consumer choice. It requires a gradual adaptation process to the salinity changes in this context. In fact, the effects of salinity changes on fish are multifaceted, encompassing osmoregulation, respiratory metabolism, the digestive system, ion homeostasis, and oxidative stress [8,9,10,11]. It inevitably involves alterations in gene expression and the activation or inhibition of signal pathways. Therefore, we need to comprehensively understand the molecular mechanisms underlying the response to salinity changes in order to carry out this domestication at the molecular level.

The gill, which directly contacts with the surrounding environment, plays an important role in osmotic pressure regulation, ion regulation, gas exchange, acid-base regulation, and nitrogen waste excretion between the external water environment and the internal blood environment [12,13,14,15]. The chloride cells within it transport ions to maintain the osmotic pressure balance within the fish body [16]. It is also a primary gas exchange organ that obtains oxygen for entry into the blood and tissues, and facilitates the excretion of carbon dioxide [12]. However, osmotic stress is a serious threat to cells and living organisms [17]. Therefore, regulating osmotic pressure is the first issue that organisms need to address when facing changes in salinity. In addition, organisms possess a certain degree of plasticity in adapting to changing environments [18,19]. It is even believed that this plasticity might contribute to evolution [20]. Here, the molecular mechanisms underlying the adaptation of a euryhaline fish (*O. melastigma*) to changes in salinity were focused on.

The *O. melastigma*, originally inhabiting seawater, is a widely salt-tolerant fish that can grow and reproduce normally in both freshwater and seawater [4,21]. It is a common model organism in marine and estuarine environments due to its small size (~3 cm for adult fish), rapid growth, short generation cycle (~3 months to sexual maturity), and simple culture and breeding process [22,23,24,25]. In the past, scholars usually researched its molecular mechanisms of response to short-term salinity changes, but rarely studied it in relation to long-term salinity changes [21,26]. Here, we investigated its physiological challenges and molecular differences in 12 h and intergenerational low-salinity stress, analyzed its adaptive molecular mechanisms, and further screened and identified a signal pathway (Hnf1ba-*slc12a1*). Specially, we validated the expression differences of *hnf1ba* and *slc12a1* genes using qPCR (quantitative real-time PCR) method, and then validated the transcriptional regulation relationship using dual-luciferase reporter assay in HEK293T cells, as well as an overexpression experiment by in vitro cultured gill cells of *O. melastigma*. Furthermore, the transcriptional binding site of Hnf1ba on *slc12a1* gene was also determined by fragment deletion and site-directed mutagenesis assays. Subsequently, we also employed CRISPR-dCas9 & Sun-Tag technology to regulate *slc12a1* expression in *O. melastigma* gill cells. By delving into the molecular mechanisms behind this remarkable trait, we are hoping to gain insights that promote low-salinity tolerance in fishes (especially in economic fishes) and contribute to the sustainable development of aquaculture and fisheries management in salt-affected water areas. These findings are of great significance for promoting the domestication of marine fish to freshwater.

## 2. Results

### 2.1. The Effects of Low-Salinity on Stress Indicators

**Figure 1:**
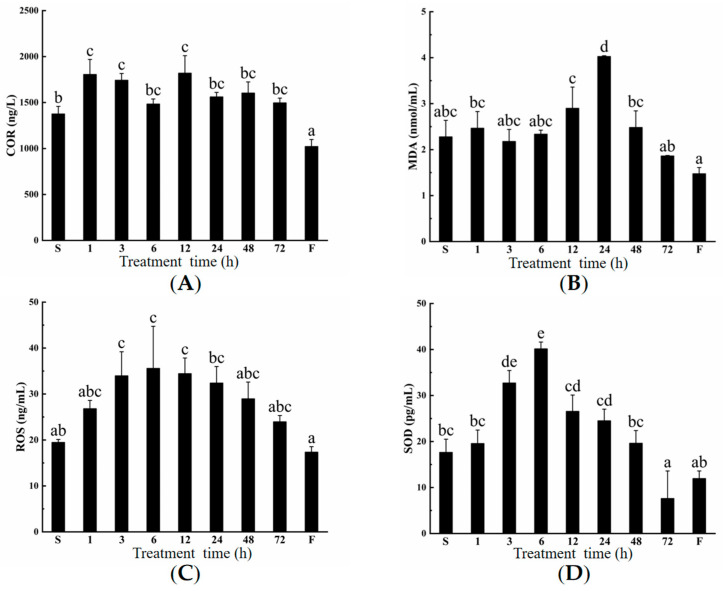
The effects of low-salinity stress on (**A**) COR, (**B**) MDA, (**C**) ROS, and (**D**) SOD in *Oryzias melastigma*. The full names of COR, MDA, ROS, and SOD are cortisol, malondialdehyde, reactive oxygen species, and superoxide dismutase, respectively. The values under the horizontal axis represent the time (h) of *O. melastigma* transferred from seawater into freshwater, while S and F represent the group of long-term aquacultures generationally in seawater (S) and freshwater (F), respectively. Different letters indicate significant differences among groups.

Stress indicators reflect the effective intensity of stress. Here, four stress indicators (COR, MDA, ROS, and SOD) were tested. After *O. melastigma* were transferred to freshwater, the COR concentration significantly increased at 1 h treatment (*p* < 0.05), and this difference was also observed in 3 h and 12 h treatment (Figure 1A). The MDA significantly increased in 24 h treatment (*p* < 0.01, Figure 1B), and there were no significant differences between the other groups and the seawater group (S). As a result of the stimulation by freshwater, the ROS concentrations showed a trend of first increasing and then decreasing overall (Figure 1C). In detail, there were significant differences compared to the seawater group at 3, 6, and 12 h treatment (*p* < 0.05). SOD also increased first and then decreased (Figure 1D). Here, it reached its peak at 6 h (*p* < 0.01), which was 2.28 times that of the seawater group. In addition, SOD of the 3 h treatment group was also significantly higher than that of the seawater group (*p* < 0.05), while that of the 72 h treatment group was significantly lower than that of the seawater group (*p* < 0.05). Therefore, considering the changes in these stress indicators, the group transferred from seawater to freshwater for 12 h was chosen as the freshwater stimulation response group for in-depth transcriptome analysis and molecular mechanism research.

### 2.2. Transcriptome Analysis

**Figure 2:**
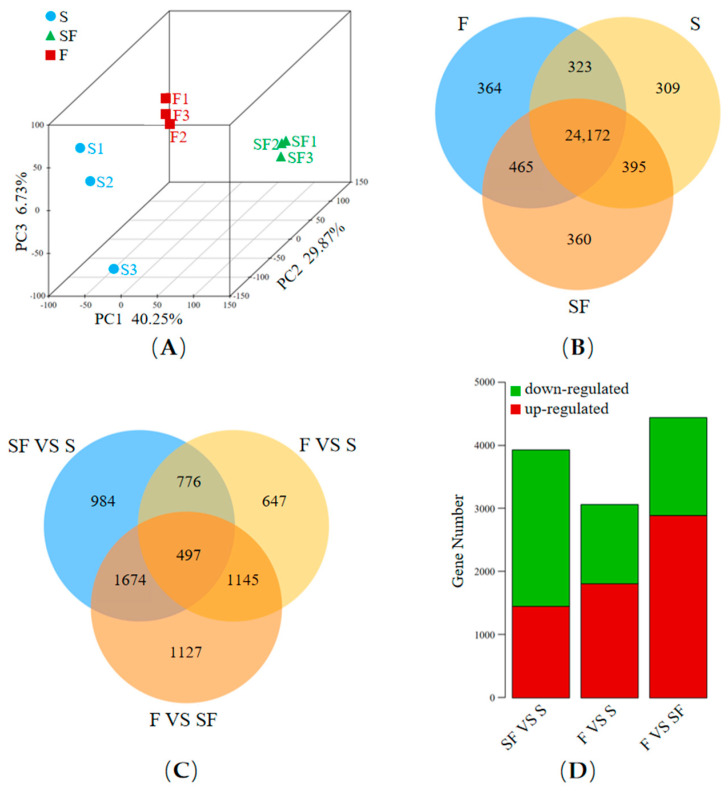
The transcriptome analysis results. (**A**) The principal component analysis at the 3D level (PCA 3D) of the samples undergoing transcriptome analysis, with S indicated by blue dots, SF by green triangles, and F by red squares. (**B**) Venn diagram showing the number of genes detected across the three groups (S, SF, and F). (**C**) Venn diagram showing the number of differentially expressed genes (DEGs) among the three comparisons: SF VS S, F VS S, and F VS SF. (**D**) The numbers of DEGs for SF VS S, F VS S, and F VS SF, respectively. “S” and “F” indicate fish grown in seawater and freshwater, respectively, while “SF” represents fish transferred from seawater to freshwater and sampled after 12 h.

We performed RNA-seq to characterize the gene expression profiles of *O. melastigma* exposed to different salinity conditions. The PCA (Figure 2A) showed a clear clustering of individuals. The three PCs explained 40.25%, 29.87%, and 6.73% of the variance, respectively. There were 25,199 genes in S, 25,392 genes in SF, and 25,324 genes in F (Figure 2B). Here, a total of 6850 DEGs (differentially expressed genes) were found across all pairwise comparisons among the three groups (Figure 2C and Supplementary Table S1). Here, the SF and F groups shared 776 genes whose expressions were significantly different from those in the S group, but these genes showed no difference in expression between the F and SF groups. A set of 497 DEGs consistently overlapped across all three pairwise comparisons (Figure 2C). The number of DEGs was 3931 (1451 up-regulated and 2480 down-regulated) in SF VS S, 3065 (1809 up-regulated and 1256 down-regulated) in F VS S, and 4443 (2891 up-regulated and 1552 down-regulated) in F VS SF (Figure 2D and Figure S1). The specific numbers of up-regulated and down-regulated genes are also shown in Figure S2A,B.

### 2.3. The Validation of Transcriptome Analysis by qPCR

**Figure 3:**
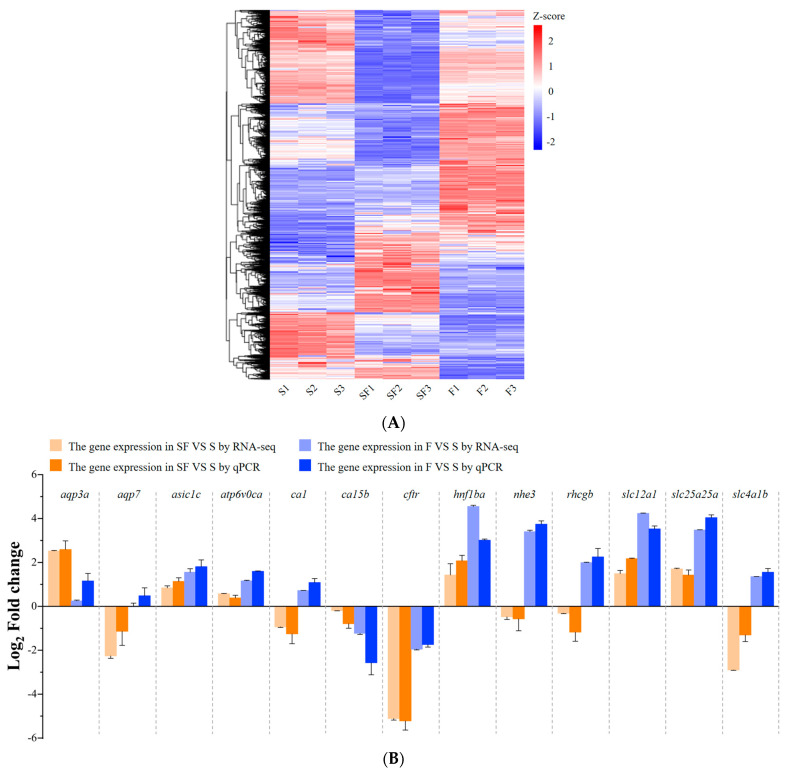
(**A**) The expressive heatmap of the DEGs in RNA-seq. The horizontal axis represents different groups, in which “S” and “F” indicate the seawater and freshwater group, respectively, and “SF” indicates the group transferred from seawater to freshwater and sampled 12 h post-transfer. The vertical axis represents the DEGs. (**B**) The expression levels of 13 genes (*aqp3a*, *aqp7*, *asic1c*, *atp6v0ca*, *ca1*, *ca15b*, *cftr*, *hnf1ba*, *nhe3*, *rhcgb*, *slc12a1*, *slc25a25a*, *slc4a1b*) were analyzed by RNA-seq and validated by qPCR.

First, the expression changes of all DEGs were examined (Figure 3A). Subsequently, 13 genes potentially involved in osmotic regulation (*aqp3a*, *aqp7*, *asic1c*, *atp6v0ca*, *ca1*, *ca15b*, *cftr*, *hnf1ba*, *nhe3*, *rhcgb*, *slc12a1*, *slc25a25a*, *slc4a1b*) were selected from the transcriptome data for qPCR validation, and the RNA-seq results were largely confirmed, with the overall expression trends of these genes broadly in agreement (Figure 3B). Notably, the target genes *hnf1ba* and *slc12a1*, together with *slc25a25a*, were consistently upregulated in both SF VS S and F VS S comparisons. This pronounced upregulation pattern suggests that these three genes may play important regulatory roles during the transition of *O. melastigma* from seawater to freshwater. Collectively, these results confirm the reliability of the RNA-seq data and provide a solid foundation for subsequent functional analyses.

### 2.4. Clustering Analysis of DEGs

**Figure 4:**
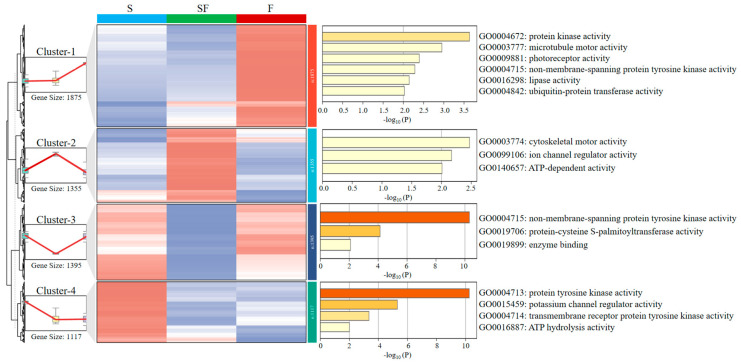
The clustering analysis results of DEGs. Cluster-1 represents genes (1875 genes) whose expression is stable from S to SF and significantly increases from SF to F. Cluster-2 (1355 genes), cluster-3 (1395 genes), and cluster-4 (1117 genes) represent genes that first rose and then fell, genes that first fell and then rose, and first fell and then remained stable during this process, respectively.

After identifying 6850 DEGs from the three comparisons (SF VS S, F VS S, and F VS SF), we conducted trend clustering analysis on the 5742 genes that had clear annotations. They were grouped into four different clusters according to the trend analysis (Figure 4, Supplementary Table S2). Cluster-1 (1875 genes) displayed an upward trend in the F group relative to the S group. In contrast, cluster-4 (1117 genes) was characterized by a downward trend across both the SF and F groups. The expression in cluster-2 (1355 genes) increased from S to SF and subsequently declined from SF to F, whereas cluster-3 (1395 genes) exhibited the opposite dynamics. GO enrichment analysis was conducted on their functions. In cluster-1, they were enriched in protein kinase activity, microtubule motor activity, photoreceptor activity, non-membrane-spanning protein tyrosine kinase activity, lipase activity, and ubiquitin-protein transferase activity. In cluster-4, they were enriched in protein tyrosine kinase activity, potassium channel regulator activity, transmembrane receptor protein tyrosine kinase activity, and ATP hydrolysis activity (Figure 4).

### 2.5. Transcription Factors and Their Target Gene Prediction

To screen for signal pathways that promoted low-salinity adaptation, the genes in cluster-1 were focused on. A total of 1349 transcription factors (TFs) of *O. melastigma* were obtained from the AnimalTFDB V4.0 (Supplementary Table S3). Among them, 88 TFs were identified in cluster-1. Based on this, the expression differences in these 88 genes among the groups were further analyzed. There were specifically 8 genes that were all significantly up-regulated in the SF VS S, F VS S, and F VS SF comparisons, including 3 members of the *hnf* (hepatocyte nuclear factor) gene family (*hnf1a*, *hnf1ba*, *hnf4a*). Among them, *hnf1ba* showed the most significant expression difference (*p* < 0.001) and was therefore selected for further research. To understand the transcriptional regulation, the target genes of the Hnf1ba transcription factor were predicted. It was reported that Hnf1b participated in osmotic pressure regulation by transcriptionally regulating members of the *slc* (solute carrier) superfamily [27]. Therefore, the expressions of *slc* gene superfamily members (455 genes) were analyzed in the transcriptome, of which 27 genes were significantly up-regulated in the pairwise comparisons of SF VS S, F VS S and F VS SF (Supplementary Table S4). In these 27 genes, the transcription binding sequences of Hnf1ba on the *slc12a1* gene promoter were predicted by JASPAR (B1: −1901~−1887, AATTAATAATTACAA; B2: −1877~−1863, GATTAATCATTTACT; B3: −1094~−1088, TGGTTAC; B4: −795~−789, TTAACTC; B5: −360~−346, GGTTAATCGTTAAGC). Subsequently, the transcriptional regulation of Hnf1ba on the *slc12a1* was verified by dual-luciferase reporter assay and overexpression experiment.

### 2.6. The Analysis and Identification of Hnf1ba Transcriptionally Regulated slc12a1 Gene

#### 2.6.1. Gene Structure and Phylogenetic Analysis

**Figure 5:**
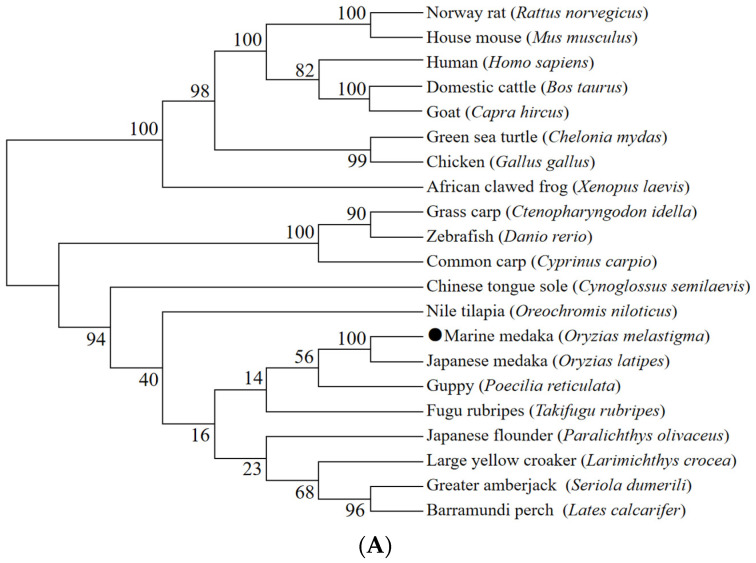

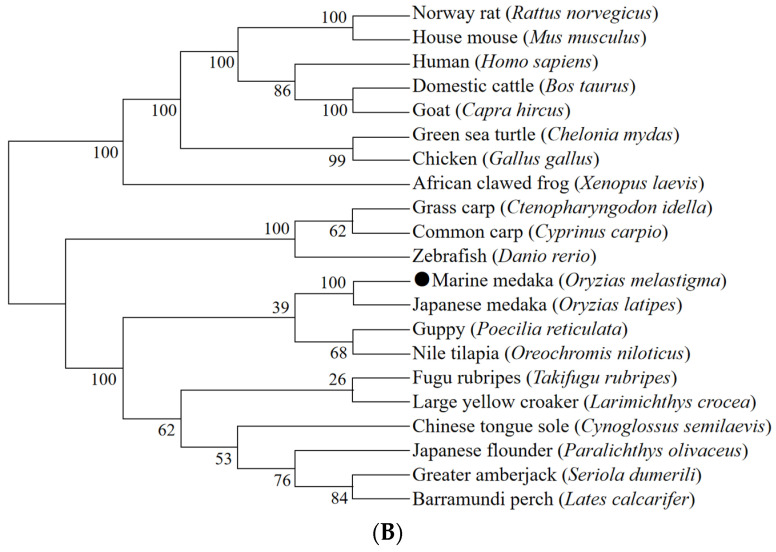
The phylogenetic trees of (**A**) Hnf1ba and (**B**) Slc12a1. The site of *O. melastigma* is marked with a solid black dot (●). The accession numbers of the Hnf1ba protein are: Marine medaka (*Oryzias melastigma*): XP_024132348.1; Zebrafish (*Danio rerio*): XP_021337152.1; Barramundi perch (*Lates calcarifer*): XP_018520922.1; Nile tilapia (*Oreochromis niloticus*): XP_005474812.1; Large yellow croaker (*Larimichthys crocea*): XP_019133362.1; Japanese flounder (*Paralichthys olivaceus*): XP_019944228.1; Greater amberjack (*Seriola dumerili*): XP_022615517.1; Japanese medaka (*Oryzias latipes*): XP_011480689.1; Chicken (*Gallus gallus*): XP_003642461.1; House mouse (*Mus musculus*): XP_006532854.1; Human (*Homo sapiens*): NP_000449.1; Guppy (*Poecilia reticulata*): XP_008423213.1; African clawed frog (*Xenopus laevis*): NP_001080685.1; Domestic cattle (*Bos taurus*): XP_005220037.1; Fugu rubripes (*Takifugu rubripes*): XP_011606849.1; Chinese tongue sole (*Cynoglossus semilaevis*): XP_024910767.1; Green sea turtle (*Chelonia mydas*): XP_037736699.1; Common carp (*Cyprinus carpio*): XP_042626969.1; Goat (*Capra hircus*): XP_017919691.1; Grass carp (*Ctenopharyngodon idella*): XP_051717912.1; Norway rat (*Rattus norvegicus*): NP_001295077.1. The accession numbers of the Slc12a1 protein are: Marine medaka (*Oryzias melastigma*): XP_024151859.1; Zebrafish (*Danio rerio*): XP_021323409.1; Barramundi perch (*Lates calcarifer*): XP_018539101.1; Nile tilapia (*Oreochromis niloticus*): XP_003456811.1; Large yellow croaker (*Larimichthys crocea*): XP_019134024.1; Japanese flounder (*Paralichthys olivaceus*): XP_019955088.1; Greater amberjack (*Seriola dumerili*): XP_022624051.1; Japanese medaka (*Oryzias latipes*): XP_004066948.1; Chicken (*Gallus gallus*): XP_413814.6; House mouse (*Mus musculus*): NP_899197.3; Human (*Homo sapiens*): NP_000329.2; Guppy (*Poecilia reticulata*): XP_008402817.1; African clawed frog (*Xenopus laevis*): XP_018108581.1; Domestic cattle (*Bos taurus*): XP_005211956.2; Fugu rubripes (*Takifugu rubripes*): XP_003969692.1; Chinese tongue sole (*Cynoglossus semilaevis*): XP_008308408.1; Green sea turtle (*Chelonia mydas*): XP_043379585.1; Common carp (*Cyprinus carpio*): XP_042631617.1; Goat (*Capra hircus*): XP_017909658.1; Grass carp (*Ctenopharyngodon idella*): XP_051726100.1; Norway rat (*Rattus norvegicus*): NP_001257546.1.

**Figure 6:**
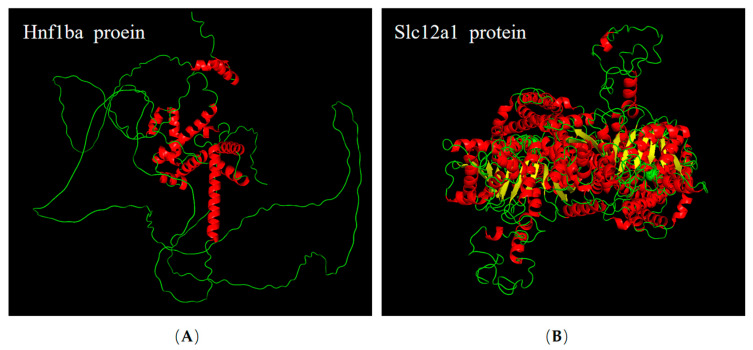
The protein three-dimensional structures of (**A**) the Hnf1ba and (**B**) the Slc12a1. The red, yellow, and green colors represent α helices, β sheets, and loops, respectively.

A comprehensive bioinformatics analysis was conducted on both proteins. The structures of these two genes (*hnf1ba* and *slc12a1*), whose exon numbers were 10 and 26, respectively, were analyzed (Figure S3A and Supplementary Table S5). In the phylogenetic analysis of Hnf1ba and Slc12a1, they were both divided into two clades (fish and other advanced vertebrates), and the two proteins of *O. melastigma* clustered more closely to those of Japanese medaka (*Oryzias latipes*) among these species (Figure 5A,B). Their protein domains were also analyzed. One homeodomain (HOX) domain were found in the Hnf1ba protein and 11 transmembrane helices in the Slc12a1 protein, respectively (Figure S3B and Supplementary Table S6). Furthermore, we also analyzed the three-dimensional structures (Figure 6A,B).

#### 2.6.2. Hnf1ba Transcriptionally Regulates *slc12a1* Gene

**Figure 7:**
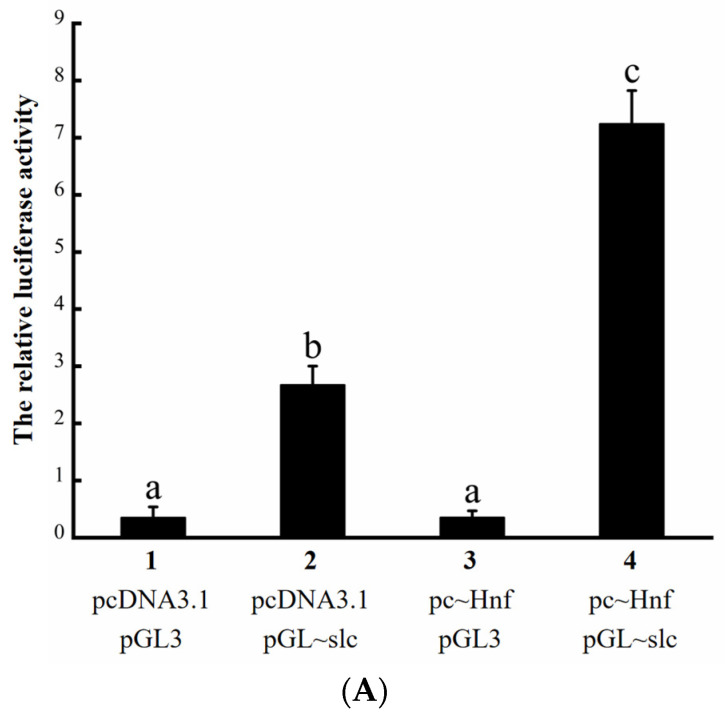

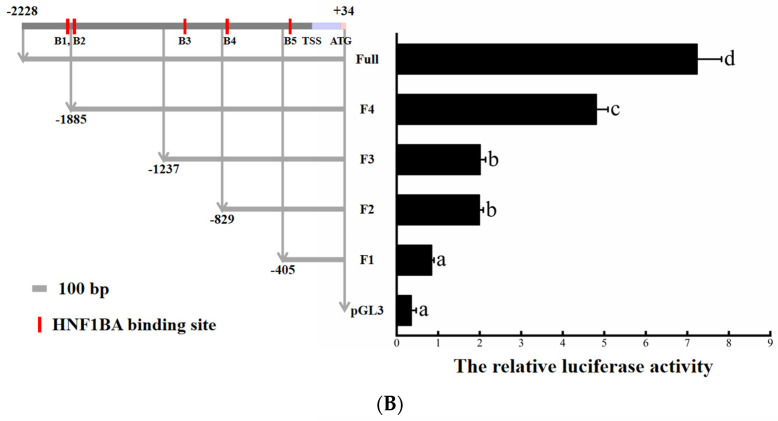

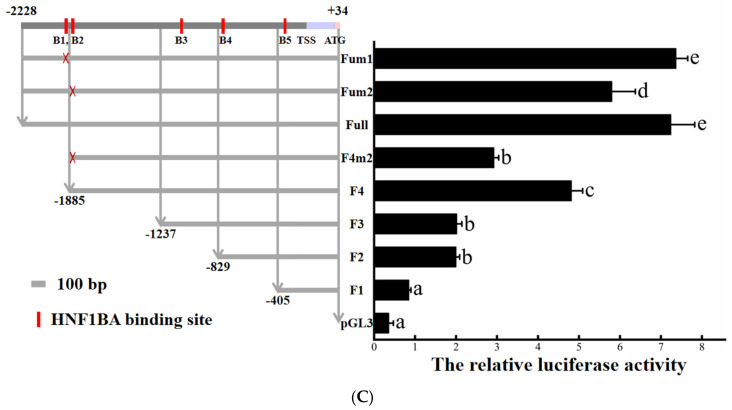
Transcriptional regulation of the *slc12a1* gene by Hnf1ba and identification of its binding sites. (**A**) Validation of Hnf1ba regulation of *slc12a1* transcription. The expression plasmids pcDNA3.1 and pc~Hnf refer to pcDNA3.1(+) vector and pcDNA3.1~Hnf1ba, respectively. Similarly, the reporter plasmids pGL3 and pGL~slc refer to pGL3-basic vector and pGL3~*slc12a1*, respectively. (**B**) The fragment deletion results of the dual-luciferase reporter assay used to identify the binding segments of Hnf1ba on the *slc12a1* gene. The five predicted binding sites are represented by B1 to B5. The pGL3, F1, F2, F3, F4, and Full represent different reporter plasmids: pGL3-basic vector, pGL3~*slc12a1* F1, pGL3~*slc12a1* F2, pGL3~*slc12a1* F3, and pGL3~*slc12a1* F4, and pGL3~*slc12a1*, each with different promoter lengths. All groups were transfected with the pcDNA3.1~Hnf1ba expression plasmid. (**C**) The site-directed mutagenesis results of the dual-luciferase reporter assay used to identify the binding sites of Hnf1ba on the *slc12a1* gene. Plasmid mutations are indicated by dark red crosses. Specifically, Fum1 represents the reporter plasmid with the mutated B1 sequence (AATTAATAATTACAA) from the Full reporter plasmid. Similarly, Fum2 is a reporter plasmid that has the B2 sequence (GATTAATCATTTACT) mutated from the Full plasmid, and F4m2 is a reporter plasmid that has the B2 sequence mutated from the F4 plasmid. All groups were transfected with the pcDNA3.1~Hnf1ba expression plasmid. Other plasmids were the same as in Figure 7B. Different letters indicate significant differences among groups.

Dual-luciferase reporter assays in HEK293T cells were used to verify the regulation of the *slc12a1* gene by Hnf1ba. The luciferase activity of the fourth group (co-transfection of pc~Hnf1ba expression plasmid and pGL~*slc12a1* reporter plasmid) was significantly higher than those of the other groups (*p* < 0.05) (Figure 7A). Notably, although the luciferase activity of the second group (co-transfection of pcDNA3.1(+) vector and pGL~*slc12a1* reporter plasmid) was less than that of the fourth group, it was significantly higher than those of the first group (co-transfection of pcDNA3.1(+) vector and pGL3 vector) and third group (co-transfection of pc~Hnf1ba expression plasmid and pGL3 vector, *p* < 0.01), likely due to basal transcriptional activity of the reporter plasmid in the absence of Hnf1ba. These results suggest that Hnf1ba may play a role in the activation of *slc12a1* transcription.

#### 2.6.3. The Transcriptional Binding Sites of Hnf1ba on *slc12a1* Gene

The fragment deletion and site-directed mutagenesis in the dual-luciferase reporter assay were conducted to identify the transcriptional binding sites of Hnf1ba on the *slc12a1* gene. In the fragment deletion experiment (Figure 7B), the first significant difference in luciferase activity occurred between F1 and F2 (*p* < 0.05), the second appeared between F3 and F4 (*p* < 0.05), and the third emerged between F4 and Full (*p* < 0.05). Five binding sites were predicted by JASPAR (Figure 7B). Among them, two predicted binding sites (B1: AATTAATAATTACAA located at −1901~−1887, B2: GATTAATCATTTACT located at −1877~−1863) were further investigated. As shown in Figure 7C, when the B2 sequence was mutated, the relative luciferase activity values of F4m2 and Fum2 were significantly lower than F4 and Full, respectively (*p* < 0.05). However, no significant difference (*p* > 0.05) was observed between the relative luciferase activity values of the Fum1 VS Full groups. These results suggest that the B2 site likely serves as a critical binding region for Hnf1ba in regulating *slc12a1* transcription.

### 2.7. The Validation of Hnf1ba-Transcriptionally Regulated slc12a1 in In Vitro Cultured Gill Cells of O. melastigma

**Figure 8:**
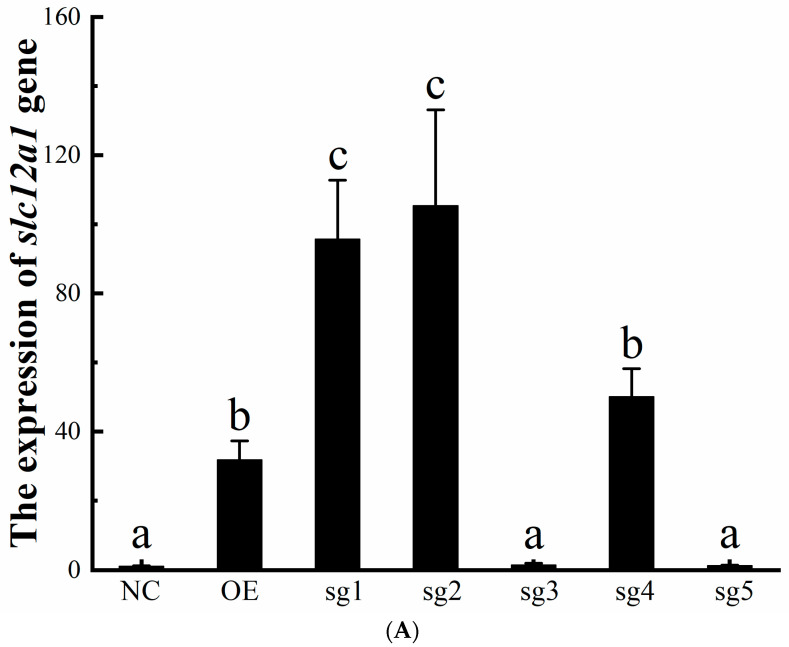

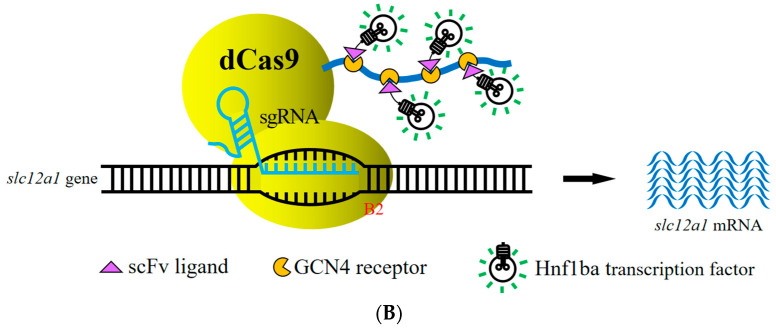
dCas9~GCN4 was co-transfected with sgRNA for targeted transcriptional activation of the *slc12a1* gene. (**A**) The expression of the *slc12a1* gene. NC represents the control group, without transfection of any plasmids. OE (overexpression) represents the group with the addition of the expression plasmid of pcDNA3.1~Hnf1ba (pc~Hnf). Sg1–5 represent groups based on the CRISPR-dCas9 & Sun-Tag technology with the addition of different sgRNA1–5 plasmids targeting the *slc12a1* gene. Different letters indicate significant differences among groups. (**B**) The experimental principle of CRISPR-dCas9 & Sun-Tag in the targeted regulation of the *slc12a1* gene by the Hnf1ba transcription factor.

To further validate the regulatory relationship between Hnf1ba and *slc12a1*, we performed targeted regulation of *slc12a1* in cultured gill cells of *O. melastigma* using CRISPR-dCas9 & Sun-Tag technology. According to the principles and requirements of sgRNA, five sgRNA sequences were designed (sgRNA1–5, Figure S4). We then co-transfected dCas9~GCN4 with the individual sgRNAs into the cultured cells and assessed the transfection efficiency by qPCR of *hnf1ba*. The results showed that *hnf1ba* expressions were significantly upregulated in all transfected groups, indicating that the co-transfection was effective (Figure S5, *p* < 0.05). The expression of the target gene *slc12a1* was significantly increased when dCas9~GCN4 was co-transfected with the sgRNA1 and sgRNA2 of *hnf1ba* respectively, which was significantly higher than that achieved by direct overexpression of *hnf1ba* (*p* < 0.05, Figure 8A,B). Notably, *slc12a1* expression was also modestly increased in the sgRNA4 than the NC group, although this change was not statistically significant in the overexpression group (OE). But the sgRNA3 or sgRNA5 groups showed no significant difference to the NC group (*p* > 0.05). Overall, these results indicate that targeted modulation of Hnf1ba may influence *slc12a1* expression, providing further support for a potential positive regulatory role of Hnf1ba.

## 3. Discussion

The *O. melastigma* is a fish native to marine environments, but it can also live normally in freshwater. To know its response to freshwater stress, stress indicators of the different treatment groups were first observed. As time passed, they (COR, MDA, ROS, and SOD) overall showed trends of first increasing and then decreasing. However, the points in time of their maximum values were different. This may be related to the responsive speed of these indicators. COR is a steroid hormone that quickly responds to stress, while MDA and ROS are related to peroxidation [28,29]. SOD is an important antioxidant enzyme that eliminates peroxidation factors in organisms effectively [30]. The stress indicator COR was lower in the F group than those in the S group. Therefore, it was speculated that these fish, having lived in freshwater for a long time, had fully adapted to the freshwater environment.

To gain a rough understanding of the functions among these 6850 DEGs, cluster analysis and GO enrichment were conducted. These analyses indicated the molecular mechanisms by which *O. melastigma* adapt to freshwater conditions as a whole. It was speculated that genes with significantly different expressions in SF compared to the other two groups (S, F) may be stress genes triggered by a sharp decrease in osmotic pressure. In fact, although many scholars have conducted studies on the effects of low-salinity on *O. melastigma*, the number of DEGs screened varies among different researches. It was reported that there were only 518 DEGs when the *O. melastigma* were transferred from seawater to 50% seawater [14]. The number of DEGs may be related to the intensity of the experimental treatment. The huge challenge faced by these *O. melastigma* was the significant change in osmotic pressure, so the factors involved in osmotic pressure regulation were further researched.

The transcription factor Hnf1ba was screened, which had a typical DNA binding domain HOX (a typical transcription factor activation domain) and was involved in the freshwater adaptation. Hnf1b was reported to affect the expression of the *slc* gene superfamily members (*slc2a1a*, *slc2a2*, *slc5a2*, and *slc5a9*) in zebrafish (*Danio rerio*) [27]. It was predicted that the *slc12a1* promoter was bound by the Hnf1ba in *O. melastigma*. Therefore, the transcriptional regulation of the *slc12a1* gene by Hnf1ba was identified. In the Hnf1ba-*slc12a1* signal pathway, their gene sequences were analyzed first, and then their protein domains and evolutionary relationships. The results indicated that Hnf1b and Slc12a1 are conserved in fish evolution. And the 11 transmembrane domains of Slc12a1 suggest a role in osmotic regulation at the cell membrane. These transmembrane domains may enhance ion reabsorption under low-salinity conditions, enabling adaptation to changes in osmotic pressure.Here, significant differences between the fourth and other groups in the dual-luciferase reporter assay demonstrated that Hnf1ba indeed regulates the *slc12a1* gene. However, it should be explained that the relative luciferase activity value of the second, which did not add the expression plasmid pc~Hnf, was significantly higher than that of the first and third. It is believed that this may be due to the regulation of endogenous factors in the HEK293 cells. In the *hnf1ba* overexpression experiment, the expression of *slc12a1* was significantly higher than that in the NC, indicating that Hnf1ba indeed transcriptionally regulates the *slc12a1* gene in *O. melastigma* cells. Furthermore, the transcription binding sites of Hnf1ba to the *slc12a1* gene were identified by fragment deletion and site-directed mutagenesis experiment. In the fragment deletion experiment, the significant difference in the relative luciferase activity values appeared between Full and F4, and between F4 and F3, indicating that the binding sequence is located between −2228 and −1885, and between −1885 and −1237. Based on the predicted results from JASPAR, mutations on the B1 sequence (AATTAATAATTACAA) and B2 sequence (GATTAATCATTTACT) were performed. The result showed no significant difference in relative luciferase activity value between Fum1 and Full, indicated that the B1 (−1901~−1887, AATTAATAATTACAA) is not a binding site for Hnf1ba. When the B2 sequence was mutated, significant differences were found between Fum2 and Full, as well as between F4m2 and F4, which indicate that the B2 (−1877~−1863, GATTAATCATTTACT) is the transcription binding site of the Hnf1ba on the *slc12a1* gene. In fact, stress response is the coordinated response of an organism as a whole, and there are also many important signal pathways that remain to be explored.

The determination of the transcription binding site provides a prerequisite for precise targeted regulating the *slc12a1* gene at an epigenetic gene editing level. CRISPR-dCas9 is a variant of CRISPR-Cas9, which has been widely used in the epigenetic gene editing field [31,32]. It loses the cleavage activity compared to the CRISPR-Cas9, but can still regulate the target gene via the fusion protein connected to dCas9 [32]. Based on this principle, specific mutagenesis of endogenous targets have been achieved by using the CRISPR-dCas9~AID (activation induced cytidine deaminase) system [33]. According to the regulatory direction of upward or downward on target genes, the system is generally divided into CRISPRa (CRISPR activation) and CRISPRi (CRISPR interference), which activate or inhibit the expression of the target gene by linking to the transcriptionally active or inhibitory element, respectively [32]. Researchers have achieved the efficient and precise regulation of their target genes (*atp6v1a*, *s100b*, *psa*) using the CRISPR-dCas9~KRAB tool, in which the KRAB (Krüppel associated box) is a common transcriptionally inhibitory element [34,35,36]. VPR (VP64-p65-Rta) is a common transcriptionally active element [37]. It has also been used in the CRISPR-dCas9 system, which has promoted the high expression of *sox2*, *oct4*, *cnga1*, *opn1mw* genes [38,39]. Furthermore, it was reported that the Sun-Tag system could improve the efficiency of regulating target genes, which was achieved by recruiting multiple active or inhibitory elements by the binding of scFv ligands to GCN4 receptors [40,41]. It was reported that researchers effectively and specifically reactivated latent *hiv-1* gene transcription using this system (CRISPR-dCas9~GCN4, scFv~VP64) [42]. Also, demethylation on the *gfap* gene was efficiently achieved using the system (CRISPR-dCas9~GCN4, scFv~TET1) [43]. Therefore, combining the confirmation of the B2 binding site, the CRISPR-dCas9 & Sun-Tag system was constructed to target the B2 binding site of the *slc12a1* gene (pcDNA3.1~dCas9~GCN4; pcDNA3.1~scFv~Hnf1ba; pGL3-U6-sgRNA-PGK-puromycin plasmids, sgRNA1–5). It has been shown that the *slc12a1* expressions were different in the five sgRNA groups, which may be related to the specificity of the sgRNA. In detail, the *slc12a1* gene expressions in the sgRNA1, sgRNA2, and sgRNA4 groups were effectively increased, especially in the sgRNA2 group, with the highest activation efficiency. It can be used to accurately and efficiently promote the targeted expression of the *slc12a1* gene in *O. melastigma*. However, the expressions of *slc12a1* in sgRNA3 and sgRNA5 were not significantly higher than those in the control group (NC), and were significantly lower than those in the overexpression experiment. It also highlights the importance of experimentally verifying efficient sgRNA for specific target genes. Overall, it is believed that the Hnf1ba-*slc12a1* signal pathway links transcriptional control to ion transport, which interacts with NKCC-mediated chloride mechanisms in *O. melastigma*. Specifically, while Slc12a1 drives Cl^−^ retention in hypotonic conditions, NKCC1 mediates Cl^−^ secretion in hypertonic environments. These effects are significant in helping fish adapt to changes in osmotic pressure.

In conclusion, the marine medaka (*O. melastigma*) was the experimental subject for studying the molecular mechanisms of freshwater domestication in marine fish. During the process of being transferred from seawater to freshwater, the *O. melastigma* exhibited gradual adaptation according to the changing trends of stress indicators (COR, MDA, ROS, and SOD). The transcriptome analysis showed that 6850 DEGs were involved in this process. By analyzing these DEGs, a signal pathway Hnf1ba-*slc12a1* was screened and subsequently identified. In detail, according to the results of the dual-luciferase reporter assay in HEK293T cells and the overexpression experiment in in vitro cultured gill cells of *O. melastigma*, Hnf1ba did promote the expression of the *slc12a1* gene transcriptionally, and this was achieved by binding to the GATTAATCATTTACT sequence (B2, located at −1877~−1863) of *slc12a1* gene. Based on this result, a targeted regulation experiment was conducted to promote *slc12a1* gene expression in in vitro cultured gill cells of *O. melastigma* by the CRISPR-dCas9 & Sun-Tag system, in which the sgRNA2 group had the highest promoting efficiency among the five sgRNA groups. These results hold great potential for the domestication of marine fish towards freshwater adaptation.

## 4. Materials and Methods

### 4.1. Fish Aquaculture and Sample Collection

All fish used in the experiment were reared in our laboratory and divided into two groups. One group was raised long-term (over generations) in seawater, and the other in freshwater. They were placed in aquariums (30 × 15 × 15 cm^3^) with filtered circulating water, where the temperature was maintained at 26 °C (26.37 ± 0.49 °C, range: 25.3–27.4 °C) throughout the year, and ammonia nitrogen levels were close to 0 mg/L (Ammonium Test Kit, Huankai Biotechnology, Guangzhou, China). And the salinity was set at approximately 30 using sea salt (Instant Ocean, Blacksburg, VA, USA) for seawater and 0 for freshwater, and was measured with a SCIONIX Salinometer (Bunnik, The Netherlands). All fish were fed twice a day (10:00 and 16:00; Tetra, Melle, Germany), and the residual bait and feces were timely removed. Here, the experimental subject was adult fish of the same age (approximately 6 months old) with an average standard length of 30.3 ± 0.6 mm, and a body weight of 550.7 ± 30.8 mg as the experimental subjects (n = 45).

Considering their original inhabitation of the marine environment, the *O. melastigma* that were in seawater were set as the control group (S, seawater), and those in freshwater for a long time (F, freshwater) and temporary time (SF, transfer from seawater to freshwater) as the experimental groups. In the SF group, the *O. melastigma* that had been living in seawater for an extended period were directly transferred into freshwater. We established seven sampling time points (1 h, 3 h, 6 h, 12 h, 24 h, 48 h, 72 h) post-transfer to freshwater. Subsequently, they were euthanized under MS-222 (100 mg/L) [44]. Then, the gill tissues were immediately placed into 1.5 mL centrifuge tubes containing TRIzol (Invitrogen, Carlsbad, CA, USA). In addition, we trimmed some muscle tissue from the caudal part (that is, the muscle which lies between the end of the anal fin or the beginning of the dorsal fin and the caudal fin) to detect stress indicators (COR, MDA, ROS, and SOD) to determine the group at the most severe stress time point, whose gill tissues were then subjected to transcriptome analysis via RNA-seq. All of these tissue samples were quickly placed in liquid nitrogen and then transferred to a −80 °C refrigerator. Additionally, a portion of the gill was used for primary cell culture. There were five biological replicates at each time point.

### 4.2. The Detection of Stress Indicators

The tail muscle tissues were fully ground in a Tissue Grinder (DHS, Tianjin, China), and then mixed with physiological saline [1 g of muscle tissue mixed with 9 mL of physiological saline] under an ice-water bath. ELISA kits designed for fish (Spbio, Wuhan, China) and the microplate reader (INFINITE F PLEX, TECAN, Männedorf, Switzerland) were used to detect the stress indicators COR, MDA, ROS, and SOD.

### 4.3. RNA Extraction and Transcriptome Analysis

The TRIzol (Invitrogen) was used to extract RNA, the concentration and integrity of which were detected by the fluorescence meter (Qubit2.0, Invitrogen, Carlsbad, CA, USA) and electrophoresis (1.5% agarose gel), respectively. Subsequently, they were used for RNA-seq and qPCR detection. In the RNA-seq, the Qubit RNA HS Assay Kit (Invitrogen) was used to accurately quantify the RNA. After mRNA purification and fragmentation, double-stranded cDNA synthesis (Hieff NGS^TM^ MaxUp Dual-mode mRNA Library Prep Kit for Illumina^®^, YEASEN, Shanghai, China), purification, end repair, dA tail addition, adapter ligation, product purification, fragment sorting, and library amplification (Hieff NGS^TM^ DNA Selection Beads, YEASEN), the cDNA library was constructed. Sequencing based on the DNBSEQ platform was conducted after quality control of the library. Clean reads were obtained by quality control (FastQC) and trimming (Trimmomatic, version 0.36) of raw reads, which were submitted to NCBI (BioProject: PRJNA1163025). Subsequently, these reads were mapped to the *O. melastigma* genome (GCF_002922805.2) using HISAT2, and the results were compared using RSeQC (version 2.6.1). The QC (quality control) results were carefully attended to, with a read depth of 9.68× and a mapping rate of 94.53%. Gene expressions were calculated by the TPM (Transcripts Per Million) method in StringTie software (version 1.3.3b). Principal component analysis (PCA) was used to visually display the similarities and differences among samples. The DEGs were analyzed by DESeq2 (version 1.12.4), in which the criteria were to set the *p* value < 0.05 and the absolute value of the log_2_ fold change (|log_2_FC|) > 1.

### 4.4. Gene Expression Validation by qPCR

To validate the confidence of the transcriptome analysis, we randomly selected 13 genes (*aqp3a*, *aqp7*, *asic1c*, *atp6v0ca*, *ca1*, *ca15b*, *cftr*, *hnf1ba*, *nhe3*, *rhcgb*, *slc12a1*, *slc25a25a*, *slc4a1b*) related to osmotic pressure regulation to detect their expression by qPCR. The RNA used in the above RNA-seq was used for reverse transcription to obtain cDNA. Subsequently, the 10 μL mixture [5 μL SYBR PCR mix (QuantiNova SYBR PCR Mix Kit, Qiagen, Hilden, Germany), 0.7 μL each for forward primer and reverse primer (Table 1), 1 μL cDNA (4 × diluted), and 2.6 μL ddH_2_O] was placed in the ROCHE Lightcycler480II (Basel, Switzerland) to conduct the qPCR experiment. Here, *18s* (18s ribosomal RNA) was used as the internal reference gene. We set triplicates of each sample as technical replicates, and calculated the relative expressions using the comparative threshold method (2^−ΔΔCt^) [45]. In addition, the WeiShengXin online platform (http://www.bioinformatics.com.cn/, accessed on 22 October 2024; China) was used to generate the cluster heatmap of gene expressions (cluster 1–4).

### 4.5. Screening of Adaptation Factors to Low-Salinity Stress

#### 4.5.1. Screen and Prediction

In order to screen the signal molecule and its pathway that promote the adaptation of *O. melastigma* to freshwater, the up-regulated genes were focused on in the SF VS S and F VS S comparisons (cluster-1). The Hnf1ba was screened for by taking the intersection of these up-regulated DEGs and the 1349 TFs set of *O. melastigma* (downloaded from https://guolab.wchscu.cn/AnimalTFDB4//#/Download (accessed on 22 October 2024), the AnimalTFDB V.0 online website). Furthermore, it was predicted that the *slc12a1* gene might be a target gene of Hnf1ba. Next, the prediction was validated.

#### 4.5.2. Gene Structure Analysis and Phylogenetic Tree Construction

The gene sequences of these two genes (*hnf1ba*, 112149117; *slc12a1*, 112161093) were analyzed by using the Gene Structure Display Server (GSDS 2.0) software (http://gsds.gao-lab.org/index.php, accessed on 17 February 2025). Their protein information (physical chemical characteristics, domains, and transmembrane helix number) was predicted by using the Expasy tool (https://web.expasy.org/protparam/, accessed on 17 February 2025), the Simple Modular Architecture Research Tool (SMART) (http://smart.embl.de/, accessed on 17 February 2025), and the TMHMM-2.0 (https://services.healthtech.dtu.dk/service.php?TMHMM-2.0, accessed on 17 February 2025). Their protein three-dimensional structures were also predicted by using the SWISS-MODEL online software (https://swissmodel.expasy.org/interactive, accessed on 19 February 2025), and were depicted in detail by the PyMOL software (version 2.5). Furthermore, phylogenetic trees for them were constructed to represent evolutionary relationships by using the Molecular Evolutionary Genetics Analysis software (MEGA 7.0) with the Neighbor-Joining (NJ) method, in which topological stability was evaluated under 1000 bootstrap replications.

### 4.6. Identification of the Hnf1ba-slc12a1 Signal Pathway

The above prediction was validated using dual-luciferase reporter assay and overexpression assay in HEK293T (human embryonic kidney 293T) cells and in in vitro cultured gill cells of *O. melastigma*. Furthermore, the transcription binding site of Hnf1ba on the *slc12a1* gene was also determined in HEK293T cells, which was utilized to accurately regulate the *slc12a1* gene through the CRISPR-dCas9 & Sun-Tag experiment in in vitro cultured gill cells of *O. melastigma*.

During the experiment to identify the signal pathway, the expression plasmid (pc~Hnf) and reporter plasmid (pGL~slc) were first constructed. In detail, the CDS (coding sequence) of the *hnf1ba* gene, obtained by high-fidelity PCR (hf-PCR; KOD One^TM^ PCR Master Mix Kit, TOYOBO, Osaka, Japan), was homologously recombined (ClonExpress Ultra One Step Cloning Kit, Vazyme, Nanjing, China) with the pcDNA3.1(+) plasmid after double enzyme digestion (HindIII and BamHI; NEB, Ipswich, MA, USA) to obtain the expression plasmid (pc~Hnf). Similarly, the reporter plasmid (pGL~slc) was also obtained by homologous recombination. The *slc12a1* gene promoter, obtained by hf-PCR, was homologously recombined with the pGL3-basic plasmid after double enzyme digestion (SacI and HindIII; NEB). Here, the primers used in hf-PCR were shown in Table 2, and their templates were cDNA and genomic DNA (TIANamp Marine Animals DNA Kit, TIANGEN, Beijing, China), respectively. In the experiment aimed at determining transcription binding sites, fragmented plasmids (F1, F2, F3, F4) and mutant plasmids (F4m2, Fum1, Fum2) were also constructed. The fragmented plasmids were also constructed by homologous recombination after the same double enzyme digestion and hf-PCR. The mutant plasmids were obtained by reverse hf-PCR, in which the F4, pGL~slc (Full), and pGL~slc (Full) were used as templates for constructing the F4m2, Fum1, and Fum2, respectively. The plasmid monoclone was selected, expanded, and sequenced by Sangon Biotech (Shanghai, China). After sequencing, these plasmids were extracted using the Endofree Mini Plasmid Kit (TIANGEN).

In the dual-luciferase reporter assay, the aforementioned plasmids were transfected into HEK293T cells. The cryopreserved HEK293T cells in liquid nitrogen were resuscitated in a 37 °C water bath. After these cells had stably grown for 3–4 generations, we put them into 24-well plates (10^5^ cells/well). Subsequently, the expression plasmid (pc~Hnf) and reporter plasmid (pGL~slc) were transfected into these cells when their confluence reached 70%–90% (Lipofectamine 3000, Invitrogen). Meanwhile, the pRL-TK plasmid (Promega, Madison, WI, USA) was used as the internal reference to characterize the transfection efficiency. After transfection for 48 h, the relative luciferase activities of firefly luciferase and Renilla luciferase were detected using the microplate reader (INFINITE F PLEX) with a Dual-Luciferase Kit (Promega, Madison, WI, USA). The ratio (firefly luciferase fluorescent value/Renilla luciferase fluorescent value) was calculated as the relative luciferase activity value (n = 3).

To verify the endogenous regulatory relationship of this pathway, overexpression experiments were conducted in in vitro cultured gill cells of *O. melastigma*. The gill cells were cultured as follows: The fresh gill tissue was quickly put into a rinse solution, which was prepared with 8% triple antibiotic solution (penicillin-streptomycin-amphotericin B solution, Solarbio, Beijing, China) and 92% DMEM medium (Solarbio). Subsequently, the gill tissue was transferred to an ultra-clean workbench for the following operations: we washed the gill tissue in a 6-well plate (NEST, Wuxi, China), chopped and shredded the gill tissue using surgical knives and scissors, and then digested the gill tissue with trypsin (Solarbio). As a result, a mixture of cells and tissue fragments were obtained. The mixture was subsequently transferred to a centrifuge tube and centrifuged for 10 min at 1500 rpm (~125 g). We removed the supernatant and dissolved the precipitate in the cell medium of *O. melastigma* (20% FBS, Gibco, Grand Island, NY, USA; 4% tertiary antibody, Solarbio; 1% embryo grinding solution of *O. melastigma*, self-made; 1% HEPES, Gibco; and DMEM medium, Solarbio). Subsequently, they were transferred to a cell culture flask (NEST) and incubated at 28 °C with 5% CO_2_. The primary gill cells of *O. melastigma* typically adhered and proliferated within one week. After growing 3–5 generations with stable status, we transferred them into a 6-well plate for transfection, the steps of which were similar to the above description in HEK293T cells. Here, the group transfected with the expression plasmid (pc~Hnf) was used as the experimental group, and the group not transfected with any plasmids as the control group. After transfection for 3 days, the cells were collected to detect the gene expressions by qPCR, which was the same as the method in Section 4.4 (Gene Expression Validation by qPCR).

In the targeted regulation experiment, the CRISPR-dCas9 & Sun-Tag technology was used to accurately target and regulate the *slc12a1* gene. Here, the plasmids (pcDNA3.1~dCas9~GCN4, Supplementary Data S1 and pcDNA3.1~scFv~Hnf1ba, Supplementary Data S2) that were obtained by modifying and splicing multiple plasmids were used to achieve the experimental aims. For the pcDNA3.1~dCas9~GCN4 plasmid, the linearized pcDNA3.1(+) was obtained by double enzyme digestion (HindIII and BamHI; NEB, USA), the dCas9~GCN4 sequence by hf-PCR using pHRdSV40-NLS-dCas9-24xGCN4_v4-NLS-P2A-BFP-dWPRE plasmid (Addgene, 60910) as the template. The pcDNA3.1~dCas9~GCN4 plasmid was obtained by homologous recombination (ClonExpress Ultra One Step Cloning Kit, Vazyme) of these two fragments. For the pcDNA3.1~scFv~Hnf1ba plasmid, the method for obtaining the linearized pcDNA3.1 was the same as described above, and the scFv sequence was obtained by hf-PCR using a pHRdSV40-scFv-GCN4-sfGFP-VP64-GB1-NLS plasmid (Addgene, 60904) as the template, and the Hnf1ba sequence by hf-PCR using a pc~Hnf plasmid (above expression plasmid) as the template. Similarly, the pcDNA3.1~scFv~Hnf1ba plasmid was obtained through homologous recombination. Here, in the CRISPR-dCas9 & Sun-Tag experiment, pGL3-U6-sgRNA-PGK-puromycin plasmid (Addgene, 51133, Supplementary Data S3) was used as the guide plasmid (sgRNA). According to the transcription binding site determined above, we designed 5 sgRNA plasmids targeting the *slc12a1* gene. These plasmids were obtained by using reverse hf-PCR, with primers listed in Table 2. The subsequent transfection and qPCR experiment for expression detection steps was the same as in the overexpression experiment. Here, all cell experiments conducted in HEK293T and gill cells were performed in triplicates.

### 4.7. Statistical Analysis

The ANOVA with Duncan’s post hoc tests under the premises of a normal distribution (Kolmogorov–Smirnov test) and homogeneity of variance (Levene test) in SPSS 22.0 software was used to analyze whether there were significant differences in the data (stress indicators, qPCR, dual-luciferase reporter assay, overexpression, the targeted regulation experiment). The *p* value < 0.05 was considered as a statistical difference, < 0.01 significant statistical difference, and < 0.001 extremely significant statistical difference. The data were presented as the mean ± standard error of the mean (M ± SEM) in the figures, which were created using originPro software (version 2018C 64-bit).

## Figures and Tables

**Table 1:** The primer sequences in the qPCR.

No.	Gene Name	Forward Primer Sequence (5′-3′)	Reverse Primer Sequence (5′-3′)	Gene ID
1	*aqp3a*	ACAAACTAGCGCGGACCTTT	CAGCACCACAGCCAAACATC	112147896
2	*aqp7*	GGGACTTGCTGAAACCCTCA	AACGACACCGCTCCATTCAT	112145787
3	*asic1c*	CTTCGGAGGTTCTCTCCCTG	GCAACAAGAAACACCGTCCA	112147473
4	*atp6v0ca*	AGAAGATAGTCTGCCCCCGT	AGCTACCACCAGCCCATAGA	112146101
5	*ca1*	GGGAATCTCAAAGCACACGC	ATGACCACCATTGGCACCTC	112151462
6	*ca15b*	GTGAAGGCGAACACATCAGC	GCGTACCGTTGATGCAAACA	112154357
7	*cftr*	CAAGACCGCAGGGACAAGAT	TCCACGAGGTCGTCACAAAG	112139207
8	*hnf1ba*	TCAGCCTGCCCACTCATTAC	AGCTGCTTCACTTCCCGTTT	112149117
9	*nhe3*	CGTGGTCTTCTTCACCGTCA	GGTCTTGCTTCTTCCTGGCT	112160835
10	*rhcgb*	TCGCCATATCGTGGATGCTC	TTGAAGCCAGCGACAGGTAG	112152785
11	*slc12a1*	CCCCCTCGGATGATTTGGAG	CTCAGGGCGATGACCACAAT	112161093
12	*slc25a25a*	ATCCTTGTGGCGAGGTAACG	GACCCGACTTTCTCAGAGCC	112137792
13	*slc4a1b*	TACATCGTTGGACGGGTGTG	CCTGGGTGAAGCGAGAGATG	112153876
14	*18s*	CCTGCGGCTTAATTTGACCC	AGTTGGTGGAGCGATTTGTC	DQ105650.1

**Table 2:** The primer sequences in the dual-luciferase reporter assay, overexpression experiment, and targeted regulation experiment.

Name	Sequence (5′-3′)	Product Length (bp)	Annealing Temperature (°C)	Gene ID
pc~Hnf	F: ctagcgtttaaacttaagcttATGTTTTCTAAAATGGTAGCCAAGC	1764	63	112149117
	R: ccacactggactagtggatccTCACCAAGCTTGAAGAGGACAC			
pGL~slc (Full)	F: ctatcgataggtaccgagctcGCACCAACTCTCTCGATGTCA	2304	63	112161093
	R: cagtaccggaatgccaagcttGCGCCCCTCTGTTAGACTTG			
F1	F: ctatcgataggtaccgagctcCAGGCAATGAGCACCTTTATCT	481	63	112161093
	R: cagtaccggaatgccaagcttGCGCCCCTCTGTTAGACTTG			
F2	F: ctatcgataggtaccgagctcCCTCAAGCATATTCCTGTAACTCA	905	63	112161093
	R: cagtaccggaatgccaagcttGCGCCCCTCTGTTAGACTTG			
F3	F: ctatcgataggtaccgagctcACCTTGACTGGAGGCTTGTTG	1313	63	112161093
	R: cagtaccggaatgccaagcttGCGCCCCTCTGTTAGACTTG			
F4	F: ctatcgataggtaccgagctcGACGTTAGGATTAATCATTTACTTTC	1961	63	112161093
	R: cagtaccggaatgccaagcttGCGCCCCTCTGTTAGACTTG			
F4m2	F: GAGCTCGACGTTAGTTCAATAATTCAAAACTGTACTTTTCTTTG	6682	60	112161093
	R: TGAACTAACGTCGAGCTCGGTACCTATCGATAG			
Fum1	F: ATAATGATAGACGTTAGGATTAATCATTTACTTTCAA	7025	60	112161093
	R: CCTAACGTCTATCATTATAAAACATTAATTTATGTCACAGC			
Fum2	F: CAAAGACGTTAGTTCAATAATTCAAAACTGTACTTTTCTTTG	7025	60	112161093
	R: ATTGAACTAACGTCTTTGTAATTATTAATTATCATTATAAA			
sgRNA1	F: accgTAACGTTCCATCTCCATCCAgttttagagctagaaatagcaagttaaaa	4945	55	112161093
	R: TGGATGGAGATGGAACGTTAcggtgtcctttccacaagatatataaagcc			
sgRNA2	F: accgGCAGGGTGTCCTTCTTTCATgttttagagctagaaatagcaagttaaaa	4945	55	112161093
	R: ATGAAAGAAGGACACCCTGCcggtgtcctttccacaagatatataaagcc			
sgRNA3	F: accgACAGGTAAACAGTAGGTGGCgttttagagctagaaatagcaagttaaaa	4945	55	112161093
	R: GCCACCTACTGTTTACCTGTcggtgtcctttccacaagatatataaagcc			
sgRNA4	F: accgTCCGCCCTTAGTTTGATCAGgttttagagctagaaatagcaagttaaaa	4945	55	112161093
	R: CTGATCAAACTAAGGGCGGAcggtgtcctttccacaagatatataaagcc			
sgRNA5	F: accgAATCATGCCGTGGATGGAGAgttttagagctagaaatagcaagttaaaa	4945	55	112161093
	R: TCTCCATCCACGGCATGATTcggtgtcctttccacaagatatataaagcc			

Note: In the primer sequences, lowercase letters represent homologous sequences on plasmids, while uppercase letters represent sequences of corresponding genes. And F and R represent forward primer and reverse primer, respectively.

## Data Availability

The RNA-seq data used in this study have been uploaded to NCBI Sequence Read Archive (SRA) with accession number PRJNA1163025. The other data will be made available on request.

## Supplementary Materials

The following supporting information can be downloaded at: https://www.mdpi.com/article/10.3390/ijms262311402/s1.
