# Supplementary material for: Screening and Identification of Hnf1ba-slc12a1 Signal Pathway in Response to Low-Salinity Stress in Marine Medaka (Oryzias melastigma)
Source: Int J Mol Sci. 2025 Nov 25;26(23):11402. doi: 10.3390/ijms262311402 (PMC12691728; doi:10.3390/ijms262311402)
Supplement: Supplementary file 1 [file ijms-26-11402-s001.zip › Supplementary figures.pdf]

Supplementary Figures

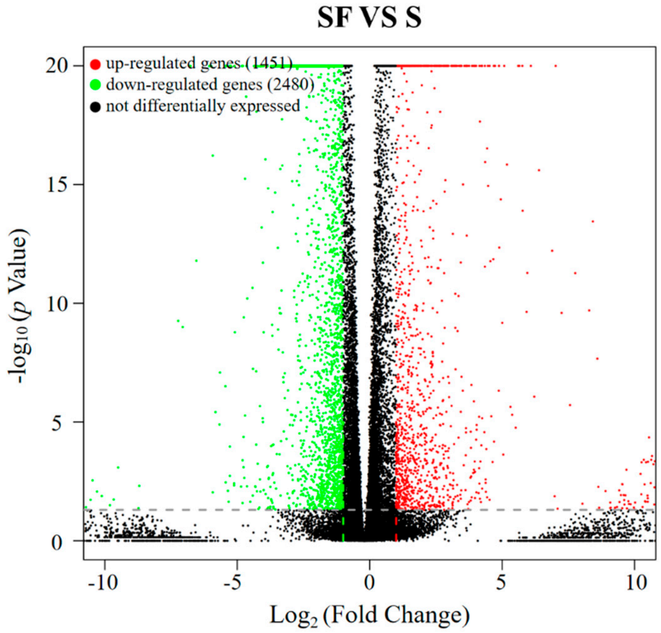

(**A**)

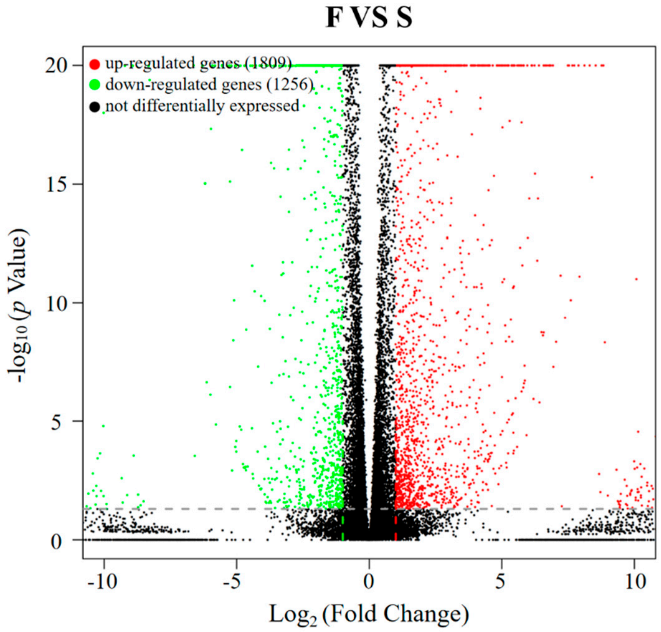

(**B**)

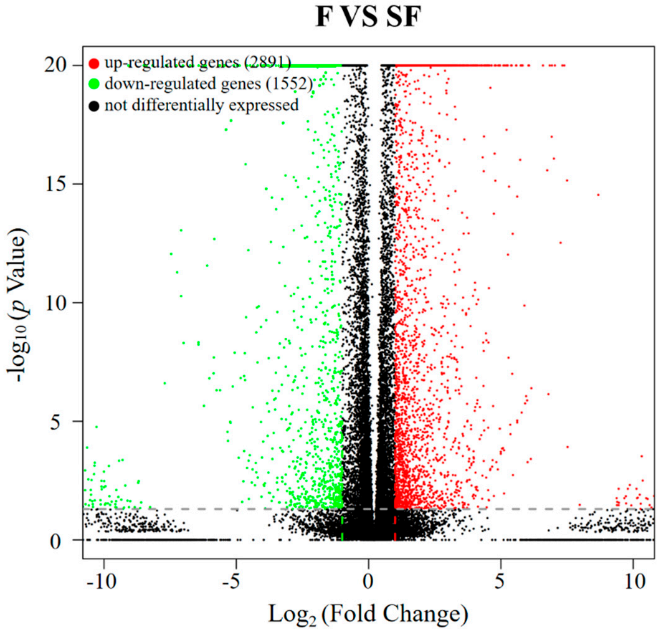

(**C**)

Figure S1. The volcano diagram of up-regulated and down-regulated differentially expressed genes (DEGs) among the three comparisons of (**A**) SF VS S; (**B**) F VS S; (**C**) F VS SF.

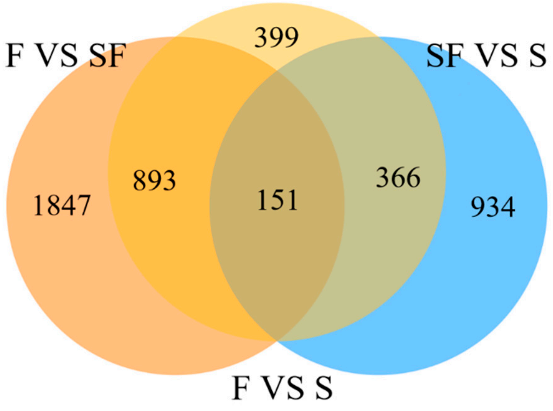

(**A**)

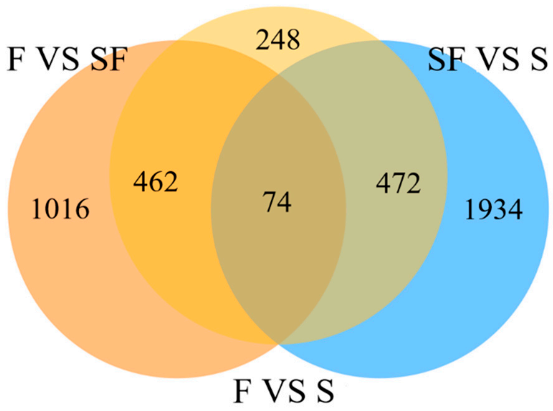

(**B**)

Figure S2. The Venn diagram of (**A**) all up-regulated DEGs and (**B**) all down-regulated DEGs among the three comparisons (SF VS S, F VS S, F VS SF).

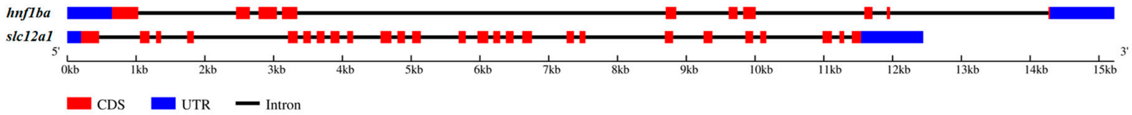

(**A**)

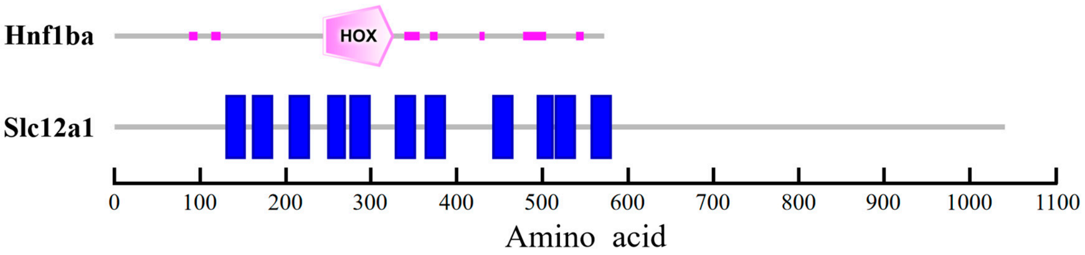

(**B**)

Figure S3. (**A**) The gene sequence analysis of the *hnf1ba* and *slc12a1* genes. The red rectangles and black lines represented CDS (coding sequence) and intron sequences, respectively. The 5′ UTR and 3′ UTR were expressed as blue rectangles on the left and right, respectively. (**B**) Domain analysis of Hnf1ba and Slc12a1 proteins. The gray lines represented the amino acid sequence of these two proteins. The magenta and blue boxes represented domains, whose names and amino acid sites (start - end) were as follows. In the 8 domains of the Hnf1ba protein, there was one homeodomain (HOX) and 7 low complexities (87 - 97, 113 - 124, 339 - 357, 369 - 378, 427 - 433, 478 - 505, 540 - 549). All 11 domains in the Slc12a1 protein were transmembrane regions: 131 - 152, 162 - 184, 205 - 227, 250 - 269, 276 - 298, 329 - 351, 364 - 386, 443 - 465, 495 - 512, 516 - 538, 558 - 580.

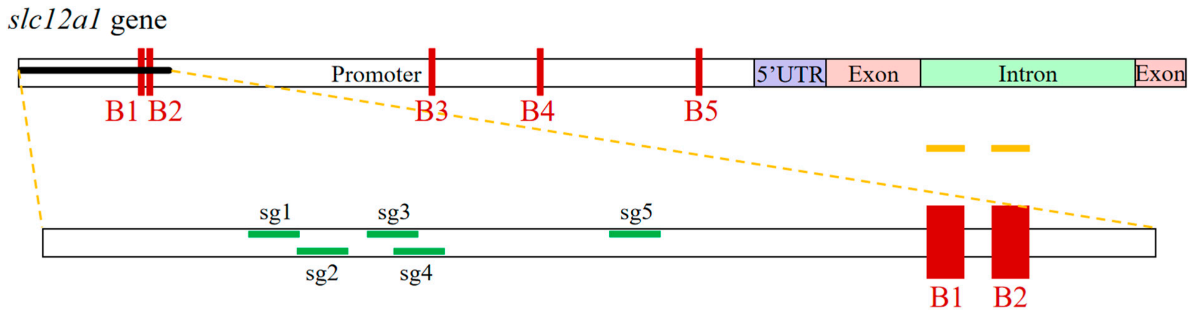

Figure S4. The 5 sgRNA targeting positions on the *slc12a1* gene. B1 – B5 represented the transcription factor binding sites for Hnf1ba on the *slc12a1* gene, predicted by the JASPAR.

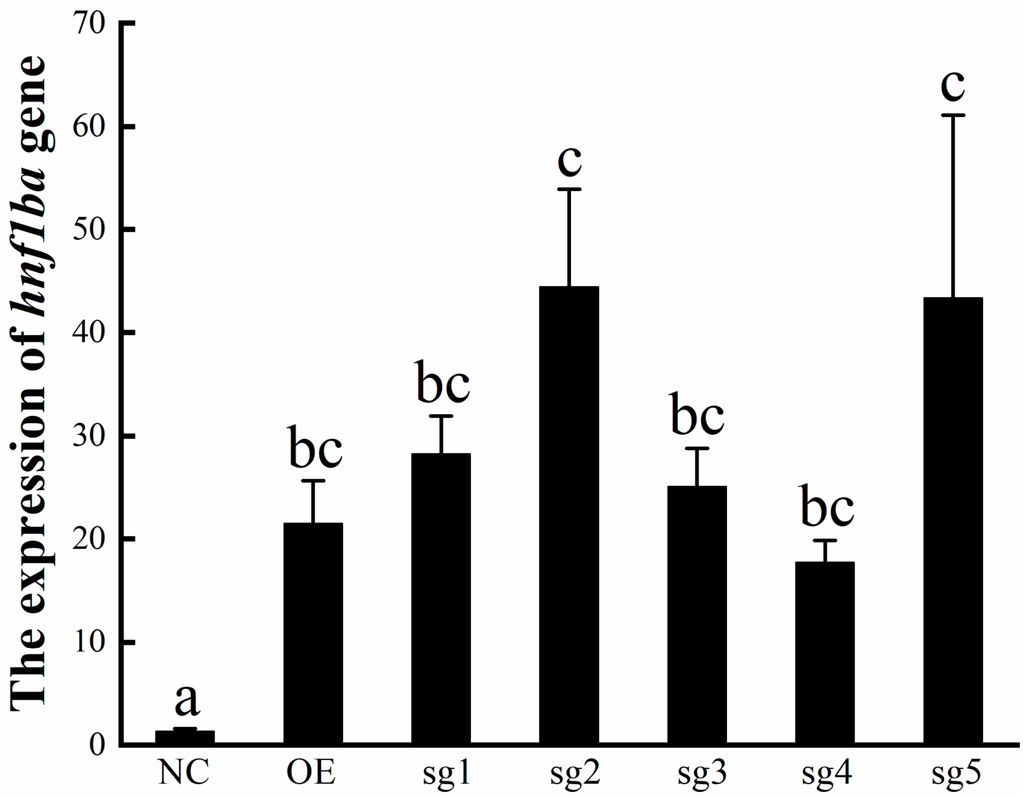

Figure S5. The *hnf1ba* gene expression in the overexpression experiment and targeted regulation experiment. NC represented the control group without adding any plasmids. OE (overexpression) represented the group with the addition of the pcDNA3.1~Hnf1ba (pc~Hnf) expression plasmid. Sg1-5 represented groups based on the CRISPR-dCas9 & Sun-Tag technology with the addition of the pcDNA3.1~dCas9~GCN4 plasmid, pcDNA3.1~scFv~Hnf1ba plasmid, and corresponding sgRNA plasmid targeting the *slc12a1* gene.
