# Supplementary material for: Screening and Identification of Hnf1ba-slc12a1 Signal Pathway in Response to Low-Salinity Stress in Marine Medaka (Oryzias melastigma)
Source: Int J Mol Sci. 2025 Nov 25;26(23):11402. doi: 10.3390/ijms262311402 (PMC12691728; doi:10.3390/ijms262311402)
Supplement: Supplementary file 1 [file ijms-26-11402-s001.zip › Supplementary Table S5&S6 Basic informationof Hnf1ba and Slc12a1.pdf]

Table S5

The gene sequence analysis of the *hnf1ba* and *slc12a1* genes.

Gene	Length (bp)	5′ UTR (bp)	Intron (number, bp)	Exon (number, bp)	3′ UTR (bp)	mRNA (bp)	Gene ID	Chromosome
hnf1ba	15,204	651	9, 11,895	10, 1722	936	3309	112149117	LG13
slc12a1	12,443	200	25, 8215	26, 3126	902	4228	112161093	LG3

Table S6

The protein sequence information of Hnf1ba and Slc12a1.

Protein	Protein length (aa)	Molecular weight (Da)	Isoelectric point (pI)	Domain (number)	Transmembrane helix	Accession number
Hnf1ba	573	62,448.92	7.13	8	0	XP_024132348.1
Slc12a1	1041	114,615.50	7.49	11	11	XP_024151859.1

The "0" in the table indicated none. Values in the column labeled "Domain" represented the number of domains. Their names and amino acid sites (start - end) were as follows. In the 8 domains of the Hnf1ba protein, there was one homeodomain (HOX) and 7 low complexities (87 - 97, 113 - 124, 339 - 357, 369 - 378, 427 - 433, 478 - 505, 540 - 549). All 11 domains in the Slc12a1 protein were transmembrane regions: 131 - 152, 162 - 184, 205 - 227, 250 - 269, 276 - 298, 329 - 351, 364 - 386, 443 - 465, 495 - 512, 516 - 538, 558 - 580.
